# Recommendations of behavioural facilitators for success in a physiotherapy clinical practice module: Successful students’ perspectives

**DOI:** 10.4102/sajp.v76i1.1392

**Published:** 2020-03-26

**Authors:** Liezel Ennion, Danelle Hess

**Affiliations:** 1 Department of Physiotherapy, Faculty of Community & Health Science, University of the Western Cape, Cape Town, South Africa

**Keywords:** physiotherapy, clinical education, student success, behavioural facilitators

## Abstract

**Background:**

Students struggle to bridge the gap between theory and application thereof in clinical settings. Exploring the behaviours of students who have been shown to be successful in the clinical practice module of physiotherapy could provide an insight into what facilitated their success. Sharing this information with other students could assist with decreasing anxiety and improving student success.

**Objectives:**

The objective of this study was to explore behaviours that facilitate student success in a physiotherapy clinical practice module from the perspective of high-achieving students.

**Method:**

Data were collected at the University of the Western Cape’s physiotherapy department in South Africa. Ten students with the highest marks in clinical practice from the 2016 and 2017 final-year cohorts were purposively selected and invited to participate in two different Nominal Group Technique (NGT) discussions. In total, 14 students consented to participate in the study. A demographic and socio-economic status questionnaire and an NGT discussion were used to collect data. Participants analysed the NGT discussion data themselves by ranking facilitators in order of priority.

**Results:**

Doing pre-block preparation, self-reflection and having a good rapport with patients as well as personal motivation and coping strategies were identified as the most important behavioural facilitators for physiotherapy students’ success in a clinical practice module.

**Conclusion:**

Clinical practice is considered to be the most stressful module for undergraduate physiotherapy students. Considering this, recommendations from previously successful students could contribute towards the success of present and future students and in decreasing the stress associated with clinical practice.

**Clinical implications:**

Recommendations from students on behavioural facilitators that enabled them to be successful in a physiotherapy clinical practice module can assist future students entering clinical practice to be successful in this stressful module. This information is also useful for clinical educators to assist students who struggle, and to recommend potential changes or improvements to the current physiotherapy clinical practice module.

## Background

Clinical education is an integral part of undergraduate physiotherapy training (Lekkas et al. 2007). Students in allied health professions struggle to bridge the gap between the theory taught in classrooms and the implementation of the same in a clinical setting (Sharif & Masoumi 2005). In a recent South African study, the clinical environment and training was identified as a major source of stress for undergraduate physiotherapy students (Janse van Vuuren, Bodenstein & Nel 2018). Similar results were reported in a study conducted on stressors amongst physiotherapy students in Pakistan (Sabih, Siddiqui & Baber 2013). Clinical practice has also been identified as the most stressful and anxiety-provoking component for students in the nursing profession (Sharif & Masoumi 2005; Sheila, Huey-Shyon & Shiowli 2002).

Bridging the knowledge–practice gap is a complex process in clinical education, and having only discipline-specific knowledge does not ensure success in the clinical environment. Factors that have assisted student learning on clinical platforms include providing opportunities to practice certain skills deliberately, such as clinical reasoning and hypothesis formulation, reflection and communication. Receiving timeous and constructive feedback has also been shown to have a positive impact on student learning in clinical settings (Ernstzen, Bitzer & Grimmer-Somers 2009).

Strategies to enhance students’ ability to clinically reason have shown contradicting results. Self-regulated learning and student interest and enjoyment have been positively correlated with academic achievements (Artino, La Rochelle & Durning 2010; Eccles & Wigfield 2002; George et al. 2008). Medical, pharmacy and nursing students report that a meaningful learning environment, interest in the subject and a positive student–supervisor relationship are important aspects for successful clinical learning (Banneheke et al. 2017); however, only limited evidence is available on student behaviours that facilitate success specifically related to clinical practice.

Some behavioural facilitators have been identified by students in other branches of health professions such as medicine, pharmacy and nursing. High-achieving medical students in Saudi Arabia report that motivational beliefs and learning strategies, such as attendance in lectures, early revision, prioritising of learning needs, learning in small groups, mind mapping and good time management, contributed to their academic achievements (Abdulghani et al. 2014). These students also noted the importance of managing non-academic factors such as stress and language barriers (Abdulghani et al. 2014). A strong association between being physically active and academic achievement was noted amongst a sample of 409 medical students at a University in Saudi Arabia (Al-Drees et al. 2016). Time management was considered to be the most important behaviour associated with undergraduate university students’ academic success across a variety of disciplines in Canada (George et al. 2008).

The current generation of physiotherapy students’ perceived behaviours in relation to their success in this particular field is underexplored and could provide valuable insight to alleviate some of the stress that students experience with clinical practice modules. Our study aimed to explore behavioural facilitators of students who have already been shown to be successful in a physiotherapy clinical practice module to improve student success in this anxiety-provoking module.

## Method

Our study was conducted at the University of the Western Cape’s (UWC) physiotherapy department in Cape Town, South Africa. A basic socio-demographic survey and Nominal Group Technique (NGT) discussion (Potter, Gordon & Hamer 2004) were utilised to collect data. Guided by the NGT methodology, 10 students who had obtained the highest marks (ranging between 68% and 82% for both cohorts) in their third-year clinical practice module at the end of 2015 and 2016 were purposively selected and invited to participate in subsequent years (2016 and 2017). A sample size of five to nine participants is considered ideal for NGT discussions (Potter et al. 2004). Eight out of 10 students contacted in 2016 (based on their final 2015 marks) agreed to participate in the first NGT discussion. Six out of 10 students contacted in 2017 (based on their final 2016 marks) consented to participate in the second NGT discussion. Data were collected from a final sample of 14 participants. Data were collected by the first author in the first term of 2016 and 2017. The second cohort was recruited to triangulate the data obtained from the first cohort to strengthen the recommendations made.

If both groups independently raised specific behavioural facilitators, it would be more likely to be applicable to their peers. Written informed consent was obtained from all participants prior to collection of data. Participants completed a basic self-developed socio-demographic survey, which also included some questions on educational history, and then participated in the NGT discussion. The NGT lasted for approximately 1 hour. One of the strengths of the NGT is that the participants are actively involved in generating and analysing the data. The procedure and process of analysis (reaching consensus through ranking) were as follows. All participants were asked individually to spend 10 min to write down the behavioural facilitators or actions they feel contributed to their success in clinical practice. Each participant then verbally shared his or her list with the rest of the group, whilst the first author documented all the suggestions on a flip-chart. After all the suggestions were documented, participants were given the opportunity to add any additional suggestions that they thought about during the sharing session. The floor was then opened to discuss all the points documented on the flip-chart, and similar suggestions were grouped into categories by the participants and duplicate suggestions were deleted. The first author then collated all suggestions with inputs from the participants into a list of 10 recommendations as decided prior to the start of the discussion. The participants then entered into another round of discussion to reach consensus on the top five behaviours by voting and ranking behavioural facilitators based on perceived order of importance. The quantitative data obtained from the basic socio-demographic survey were analysed descriptively.

### Ethical consideration

Ethical clearance to conduct this study was obtained from the University of the Western Cape’s senate research ethics committee (No. 15/7/101), the registrar of the University and the head of the Physiotherapy Department.

## Results

The mean age of participants in the 2016 cohort was 21 years and 9 months (range 21–24 years), and for the 2017 cohort, it was 21 years and 8 months (range 21–23 years) (Table 1).

**TABLE 1:** Overview of socio-demographic variables of both cohorts.

Cohort	Age		Gender		Final secondary school mark		Final mark for clinical practice module	
	Mean	Range	Female	Male	Average (%)	Range (%)	Average (%)	Range (%)
2016 (*N* = 8)	21 years 9 months	21–24	6	2	72.6	66–76	77.2	67–84
2017 (*N* = 6)	21 years 8 months	21–23	6	0	73.6	70–82	71.1	68–74

In the 2016 cohort, five out of eight participants resided at home, and four out of six of the 2017 cohort resided at home. Only two out of eight in the first cohort worked on a part-time basis, whilst four out of six in the second cohort worked on a part-time basis. The average mark for the clinical practice module for the 2016 cohort was 73.6% (range 70% – 82%), whilst the average for the 2017 cohort was slightly lower at 71.1% (range 68% – 74%).

In the 2016 cohort, three out of eight participants’ parents had a university education, whilst only one out of six participants in the second cohort had a parent with tertiary qualification.

The top five facilitators to success in the clinical practice module were ranked in order of importance and documented for each of the two NGT groups (Table 2). Both groups reached consensus that *doing pre-block and daily preparation* was the single most important facilitator for success in clinical practice. Both groups included *regular self-reflection* and *having a good rapport with patients* as part of the top five behavioural facilitators for their success.

**TABLE 2:** Nominal group technique discussions’ results according to ranked importance.

2016 Cohort	2017 Cohort
1. Pre-block and daily preparation	1. Pre-block and daily preparation
2. Peer learning and sharing of experiences	2. Personal factors (internal motivation)
3. Self-reflection	3. Having good coping strategies
4. Having a good rapport with patients	4. Having a good rapport with patients
5. Enjoying what you do	5. Self-reflection

## Discussion

### Pre-block and daily preparation

Diligent preparation was the most important behavioural strategy that was raised by both cohorts. Participants from both groups unanimously agreed that being adequately prepared prior to starting a new clinical rotation and daily preparation was important for their success. Self-empowerment through preparation was also highlighted by students in a similar study exploring factors that led to student success at a community college in the United States (Martin, Galentino & Townsend 2014). The same sentiment was echoed by nursing educators (Lewallen & DeBrew 2012). Furthermore, similar learning strategies, such as attendance in lectures, early revision and prioritising of learning needs, were also suggested by high-achieving medical students in Saudi Arabia (Abdulghani et al. 2014). Currently, pre-clinical block theory preparation is being encouraged at UWC’s physiotherapy department, requiring all students to pass a theory test based on specific clinical settings prior to entering a new clinical area. However, this alone does not seem to be sufficient to ensure success in the clinical block. The intrinsic motivation to continue learning whilst in clinical settings seems to have a bigger impact on success as measured by clinical examination. From the students’ perspective, daily preparation and engagement with clinical conditions and supervisors that students encounter whilst on the block are more valuable in terms of ensuring success. During the discussion on this point, students placed specific emphasis on the necessity of doing daily research on the conditions and unusual diagnoses that they came across during the day and making use of online resources for demonstrations and revision of clinical skills. Both cohorts emphasised the need to engage actively during clinical rotations. Active engagement in classrooms (or clinical site) is widely recognised as a strategy for success in higher education (Tinto 2012).

The more the student is interested, the more she or he engages in the subject (Pekrun 2006). Student suggestions for active engagement during a clinical block included writing down notes during the day, consulting and discussing patients with clinicians and asking questions. Accordingly, the authors recommend that more emphasis must be placed on developing strategies to encourage and monitor student engagement during their clinical rotations.

### Self-reflection

There is a known positive relationship between physiotherapy students’ ability to reflect critically on their clinical experiences and their clinical practice performance (Brooks et al. 2017). In our study, both cohorts highlighted the importance and value of constant self-reflection on their own weaknesses and actively looking for areas of improvement. For both cohorts of students, feedback from (clinical) supervisors and clinicians was considered important to identify and reflect upon the areas of improvement, indicating that the practice of reflection is a behaviour that needs to be taught or facilitated. The student is dependent on effective feedback from clinical educators to identify and reflect upon the areas of improvement. Without adequate feedback, students struggle to improve their clinical skills (Lewallen & DeBrew 2012).

This finding is however contradicting the findings of another South African study where only 17% of the participating physiotherapy students felt that reflection was a learning opportunity from which they greatly benefitted (Ernstzen et al. 2009). This contradiction might be explained if students are not familiar with the practice of reflection, as it does not come intuitively (Rowe 2012) and needs to be facilitated.

In Tharp and Gallimore’s (1991) theory of assisted performance, they propose that students learn from those (educators) who know more than the students through different contextualised activities based on actual experience.

These learning activities can include modelling of desired behaviour, providing feedback and correcting student activity to stimulate thoughtful responses and reflection through questioning. Similarly, in the South African study conducted by Ernstzen et al. (2009), 93% of students identified demonstration by clinical supervisors as a valuable learning activity. Students struggle to bridge the knowledge–practice gap by themselves (Rowe 2012) and to identify and reflect on their own weaknesses (Rowe 2012). The role of critical reflection in the success of clinical practice is clear, and clinical supervisors should assist students to practice reflection. However, educators themselves are not necessarily skilled in the practice of reflection, and clinical educators must be equipped in the art and process of self-reflection to be able to model it for students.

### Good rapport with patients

There is a general decline in students’ ability to communicate well and actively listen to patients (Conn et al. 2012). In the South African context, language barriers often play a hindering role, but lack of empathy or inability to ‘enter into a patient’s shoes’ are also highlighted in the literature as a limiting factor, especially during clinical examination (Cundell 2017).

In our study, students considered ‘having a good rapport with patients’ a vital facilitator to their success in the clinical practice module. There is consensus in the literature that empathy is an asset and essential skill for healthcare providers to build a good rapport with patients (Cundell 2017; Pohontsch et al. 2018; Williams et al. 2014).

Empathy is also considered central for effective healthcare provision, improved patient outcomes and continuity of care (Cundell 2017; Pohontsch et al. 2018; Williams et al. 2014). If empathy is essential for good patient rapport, then the question arises, can this be taught? There is, however, an ongoing debate whether it is a cognitive attribute, which could be learnt, or a personality trait, and if it could actually be evaluated (Cundell 2017; Sulzer, Feinstein & Wendland 2016; Williams et al. 2014). A study conducted with undergraduate nursing students, involving 10 weeks of 2-h lectures on empathy suggested that it could successfully be taught (Ozcan, Oflaz & Bakir 2012). The challenge is posed to educators to incorporate this often neglected soft skill into the health professions curriculum.

Apart from incorporating empathy and communication skills into the curriculum, Dang et al. (2017) provide some additional suggestions for health professionals for building early rapport with patients. In their study on building patient trust and rapport, they conducted interviews with patients who were enrolled in a human immunodeficiency virus programme. From these interviews, five practical suggestions were deduced from the patients themselves on how to build trust and patient rapport. These included: (1) providing reassurance to patients, (2) telling patients that it is okay to ask questions, (3) interpreting and explaining medical test results to patients, (4) avoiding language and behaviours that are judgmental of patients and (5) asking patients what they want from the treatment. If empathy could be taught or learnt, then healthcare educators could use these suggestions and similar strategies to teach this elusive skill to future healthcare professionals. This not only will benefit the students in being more successful but will also result in better patient outcomes.

### Sharing experiences

Learning has long been understood to be a social more than an individual endeavour (Seely-Brown, Collins & Duguid 1989). Sharing of experiences with colleagues develops critical reflective thinking, which, in turn, assists with clinical reasoning (Welch & Dawson 2006). Peer-assisted learning has become increasingly popular in health profession’s education to cope with increasing student numbers, but it also benefits student learning in a clinical setting (Sevenhuysen et al. 2015). Peer-assisted learning is reported to reduce students’ anxiety in clinical settings and improve their confidence and communication skills (Sevenhuysen et al. 2015). The 2016 cohort in our study felt that making use of peer learning and sharing of experiences facilitated their understanding of clinical situations and assisted them to do well in their assessment tasks. The same sentiment was not echoed by the 2017 cohort. These facilitators to success were also contradicted by physiotherapy students in an earlier study conducted by Löfmark and Wikblad (2001), where only 33% rated ‘group learning sessions’ as valuable learning opportunities. However, participants in the same study rated ‘discussions with the teacher’ as a more important learning opportunity (Löfmark & Wikblad 2001). Based on these findings and discrepancies in the literature (Williams & Reddy 2016) on the outcome of peer-assisted learning on academic achievement, no blanket recommendation could be made. Peer-assisted learning could never become a substitute to clinical skills and experience of the educator but could be useful to develop other skills that might contribute to success, beside the traditional model of teacher–student learning (Sevenhuysen et al. 2015). Students learn differently. Whilst some students prefer learning in a group, others prefer self-directed learning. For the majority of participants our study, self-directed learning was important to success.

### Having good coping mechanisms

University students are increasingly stressed and anxious (Galante et al. 2018; Janse van Vuuren et al. 2018). Clinical practice is a large component of all medical courses at the university level and adds another dimension of stress over and above the normal academic pressure (Chan, So & Fong 2009; Janse van Vuuren et al. 2018).

Having good coping strategies is necessary to do well in clinical practice, especially during exams. The majority of participants in one of the cohorts reported that they rely on anxiolytics to cope with the stress of clinical exams. Some participants advocated for regular physical exercise to cope with stress, whilst others recommended visualisation techniques prior to exam, or ‘zoning out’ the examiner. All participants agreed that just ‘listening to the patients’ also helped them succeed in the exam.

The need for interventions aimed at reducing stress amongst students is clear. Main stressors are to be considered to reduce stress. In our study, the clinical exam was reported to be the biggest cause of stress.

When considering similar studies in the literature, nursing students (Chan et al. 2009) suggest a ‘lack of professional knowledge and skills’ as their most common stressor during clinical practice, whilst ‘suffering and death, academic pressure and tension during interaction with qualified personnel’ were the stressors mentioned by physiotherapy students (Janse van Vuuren et al. 2018). Suffering and death of patients were the main cause of stress in the latter group of students. Chan et al. (2009) used the Coping Behaviour Inventory (CBI; Sheu et al. 1997) to identify nursing students’ coping strategies with examination stress. The CBI lists the following four types of coping strategies: ‘avoidance behaviours’ (efforts to avoid the stressful situation), ‘problem-solving behaviours’ (efforts to manage or change the stress arising out of a stressful situation), ‘optimistic coping behaviours’ (efforts to keep a positive attitude towards the stressful situation) and ‘transference behaviours’ (efforts to transfer one’s attention from the stressful situation to other things). ‘Transference’ was identified as the most popular coping strategy in this cohort (Chan et al. 2009). This was echoed in our study where one of the coping strategies mentioned by students was to pretend that the examiner was not there. In contrast, physiotherapy students in the study by Janse van Vuuren et al. (2018) emphasise ‘talking to someone’ (a friend or family member) as a dominant coping strategy in a stressful situation. Janse van Vuuren et al. (2018) iterate the need for student support during pre-clinical and clinical years to assist students to cope with stress. Some suggestions to provide student support include peer tutoring, academic-support lecturers and dual mentoring (peer and academic mentoring) (Janse van Vuuren et al. 2018). Communication platforms managed by qualified personnel and debriefing are additional methods used in the clinical years to support students (Janse van Vuuren et al. 2018). Similar to what was suggested by some of the participants in our study, the literature also supports cognitive, behavioural and mindfulness exercises to counter university stress (Donaghy & Morss 2007). Physical activity is also helpful to manage stress and supports academic achievement (Al-Drees et al. 2016).

Some of the above interventions could potentially be introduced to the curriculum of a clinical practice module as an alternative to medication to assist students who experience high levels of anxiety.

### Internal motivation and enjoying what you do

In spite of the general precedent that good marks in secondary school do not necessarily predict success in clinical practice (Artino et al. 2010; Cundell 2017), all students of this high-performing cohort had achieved high marks in school examinations. This association between high marks and success in clinical practice could possibly be explained by the fact that both these cohorts of students seem to be internally driven to succeed, as evidenced by their recommendations for success. The fact that both cohorts recommended diligent preparation, self-reflection and being internally motivated (either by success or a passion for what you do) speak of a strong sense of self-regulation that contributes to success in most aspects of life (Weinstein, Husman & Dierking 2000). The literature supports the notion that internal motivation and emotions, such as enjoyment of achievement, are strong contributors to success in health profession education (Artino et al. 2010; George et al. 2008). Eccles and Wigfield (2002) as cited in Artino et al. (2010) argue that the perceived interest and value that a student places on a particular educational task or course affect the student’s motivation to succeed at it. Pekrun (2006) in the ‘control-value theory of achievement emotions’ supported this, and proposed that if an educational course or task is considered interesting and valuable, students are more likely to attach emotions to enjoy it and academically perform well in it. The converse is also true, that is, if the course is considered boring, and assessments cause anxiety, students are less likely to perform well (Pekrun 2006). This places the onus on the educators to contextualise activities for students in order to emphasise the value of clinical practice.

This might stimulate students’ interest and enjoyment in the module, assisting them to succeed. Furthermore, educators should make every effort to ensure that students are actively engaged during lectures and clinical rotations to stimulate motivation and pleasure.

## Limitations and recommendations

Owing to the small sample size, the findings of this study are to be interpreted with caution. Even though the students’ recommendations could potentially be beneficial for all undergraduate physiotherapy students who enter clinical practice, the findings cannot be generalised because of the nature of qualitative methods and the fact that data were collected only from one institution.

The facilitators highlighted in our study could assist undergraduate students to adopt some of the mentioned practices in order to be more successful in a clinical practice module. Pre-clinical rotation preparation, self-reflection and having a good rapport with patients were the most important behavioural facilitators reported by this high-achieving cohort of students. Coping strategies and personal motivation were also highlighted as facilitators to success. These recommendations made by successful third-year physiotherapy students could potentially be useful to guide other students in improving their performance in the physiotherapy clinical practice module.

## Conclusion

Participants in this study identified several recommendations for succeeding in one of the most stressful modules of the physiotherapy programme. The majority of these recommendations were supported by the educational literature from related fields such as medicine and nursing. Physiotherapy clinical educators could use the suggestions of these students combined with some suggestions highlighted in the literature for the success of their students in their clinical interactions and examinations. Coping mechanisms to support students who perceive clinical practice as extremely stressful should also be included as part of the curriculum.
